# Five-Year Recall after Treatment of External Cervical Resorption

**DOI:** 10.1155/2019/4957408

**Published:** 2019-12-19

**Authors:** Fahd A. Aljarbou

**Affiliations:** Department of Restorative Dental Sciences, Division of Endodontics, College of Dentistry, King Saud University, Riyadh, Saudi Arabia

## Abstract

The management of external cervical resorption can be challenging. This study described a two-step procedure for the treatment of external cervical resorption. It involved nonsurgical root canal treatment and a dental amalgam core buildup, followed by a surgical approach to restore the root structure using resin-modified glass ionomer (Geristore®). The patient was recalled for a period of five years, and the overall outcome was successful.

## 1. Introduction

Root resorption is a physiological process in primary teeth and a pathological process in permanent teeth. It is a rare and undesirable occurrence in permanent roots. On the contrary, bone undergoes constant remodeling, including periods of resorption and deposition [1]. The process of root resorption involves two major events: injury to the protective dental tissue and activation of an inflammatory response [2]. It can affect the internal or external part of the root. It may be transient or progressive [3]. External cervical resorption (ECR) is an example of progressive external root resorption. It is been studied extensively by Heithersay, who termed it as invasive cervical resorption (ICR) [4, 5]. The defect appears extensive clinically and occurs at the cervical third of the root, possibly with a pinkish discoloration of the crown. It is classified into four classes depending on its severity, in which the more the class, the worse in clinical outcome it gets. The aim of this study was to report the management of a case of ECR involving the maxillary left canine.

## 2. Case Report

A 70-year-old man of European descent, with controlled type II diabetes, was referred to us by the periodontics clinic. He took 81 mL of aspirin orally, once a day, and 10 mEq of potassium citrate orally, thrice a day. He had no known allergies to dental materials. The patient was asymptomatic. He was referred to us, after the intraoperative observation of resorption in the palatal half of the coronal one-third of the root of tooth #23, during periodontal surgery related to an implant placed in the region of tooth #24. The invasive resorbing tissues were deep and confined to the coronal third and it is classified as Heithersay class III. Root repair was attempted using Fuji IX GIC (GC Corp, Japan), with poor results. Tooth number 23 was an abutment in joint crowns of multiple units, extending from canine to canine. Informed consent was obtained upon the patient's arrival. The cold test was normal for 22 and was negative for 23. Percussion and palpation were normal in this area. Palatal recession, measuring 2 mm, was observed with 23. The probing depth was within 2-3 mm on the buccal aspect and 7-8 mm on palatal aspect, which felt soft on tactile perception, along the inner part of the tooth indicating the presence of granulation tissue and confirming the diagnosis of ECR. Nonsurgical root canal treatment was planned, followed by surgical repair of the defect.

One carpule of 1.8 mL solution of lidocaine 2% with 1 : 80,000 epinephrine (Lidocaine HCl, Huons Co., Seoul, Korea) was used for buccal and palatal infiltration anesthesia for 23. Rubber dam was used to isolate the operating field. An access opening (into the canal) was created, using a long shank round-ended tapered diamond bur at a 45° angle for about 4 mm, while preserving the lingual triangle. Isolation was difficult to achieve, and electronic determination of working length was not feasible. The working length (29 mm) was obtained radiographically, using size 15 K-hand file (Dentsply Co., Munich, Germany) from the cusp tip. Rotary instrumentation was performed up to size 35, using the entire sequence of ProTaper® Universal (Dentsply Tulsa Dental Specialties, Tulsa, OK, USA) up to size F3, followed by ProFile Ni-Ti files (size: 35, 0.04 taper) (Maillefer, Ballaigues, Switzerland). Irrigation was performed with 12 cc of 5.25% sodium hypochlorite between each file and as a final rinse after 17% EDTA (5 mL) (Meta Biomed Co. Ltd., Mandaluyong, Korea). The canal was dried with sterile paper points and obturated with the warm vertical compaction technique, 3 mm short of the apical margin of the resorption, using Kerr Pulp Canal Sealer EWT (Kerr, Orange, CA) and gutta-percha cones (size: 35, 0.04 taper) (Meta Biomed Co. Ltd., Cheongju City, Chungbuk, Korea). The palatal access opening in the crowned tooth was filled with dental amalgam (Valliant ™, Ivoclar Vivadent Inc., Amherst NY) and vertically adapted using a range of Schilder pluggers (Dentsply, Maillefer, Switzerland).

**Figure 1:**
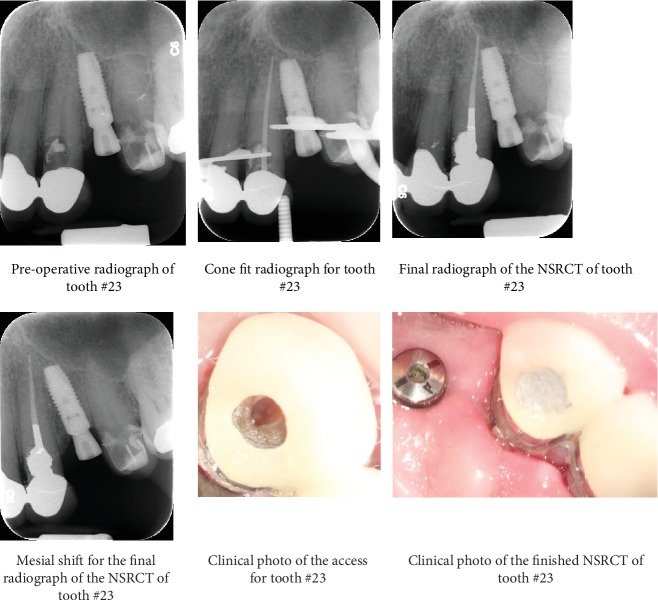
Radiographs and clinical photos showing the steps of nonsurgical root canal treatment of tooth #23 with amalgam post and core buildup.

**Figure 2:**
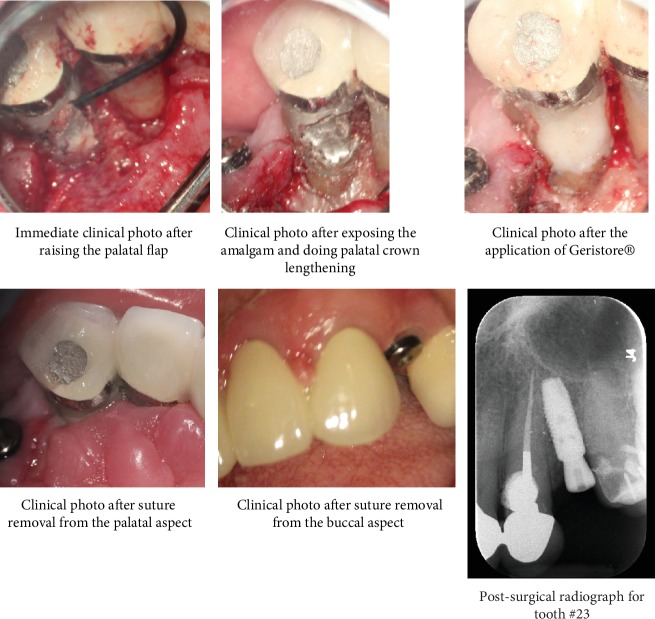
Clinical photos and postsurgical radiograph showing the whole steps for managing the resorptive defect surgically in tooth #23.

**Figure 3:**
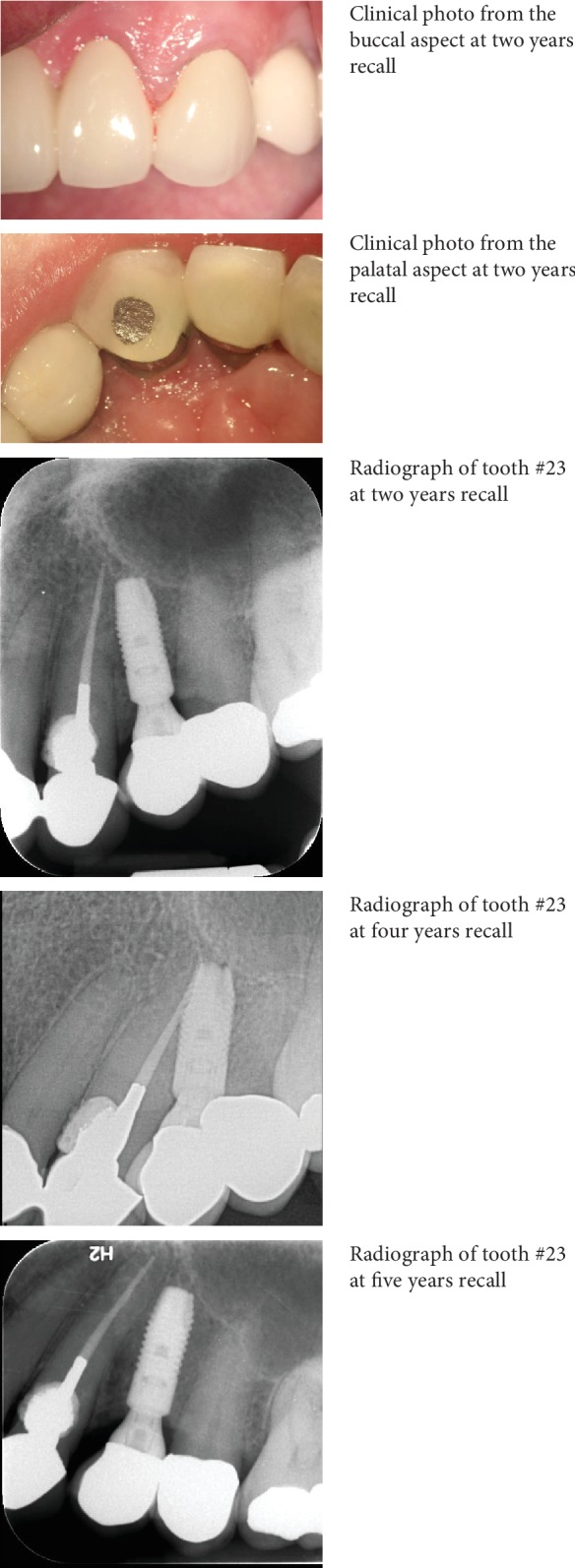
Recall radiographs and clinical photos for tooth #23.

The patient was reassessed a week later, and no significant changes were observed. Surgery was confined to the palatal aspect and two carpules containing 3.6 mL of 2% lidocaine with 1 : 100,000 epinephrine (Lidocaine HCl, Huons Co., Seoul, Korea) were used for buccal and palatal infiltration anesthesia for 23. A triangular flap was raised, subsequent to a palatal intersulcular incision from 21 to 23, with 1 cm distal vertical release. The surgical site was exposed, and the poorly adapted repair material was removed, followed by crown lengthening along the palatal margin. Geristore® (Den-Mat, Santa Maria, CA) was used to cover the fully set dental amalgam, to preserve the palatal root contour, and enhance gingival tissue attachment. Finishing and polishing were followed by five simple interrupted sutures using silk (size 5-0, reverse cutting needle FS-2, Ethicon Inc., Somerville, NJ, USA). Postoperative instructions were given and suture removal was scheduled after three days. The patient was scheduled for a proper follow-up visits in the future.  Figure 1 represents the radiographic and clinical photographs of the nonsurgical management of 23.  Figure 2 represents the clinical photos of the surgical management in 23.  Figure 3 shows the recall radiographs and clinical photographs for 23.

## 3. Discussion

**Figure 4:**
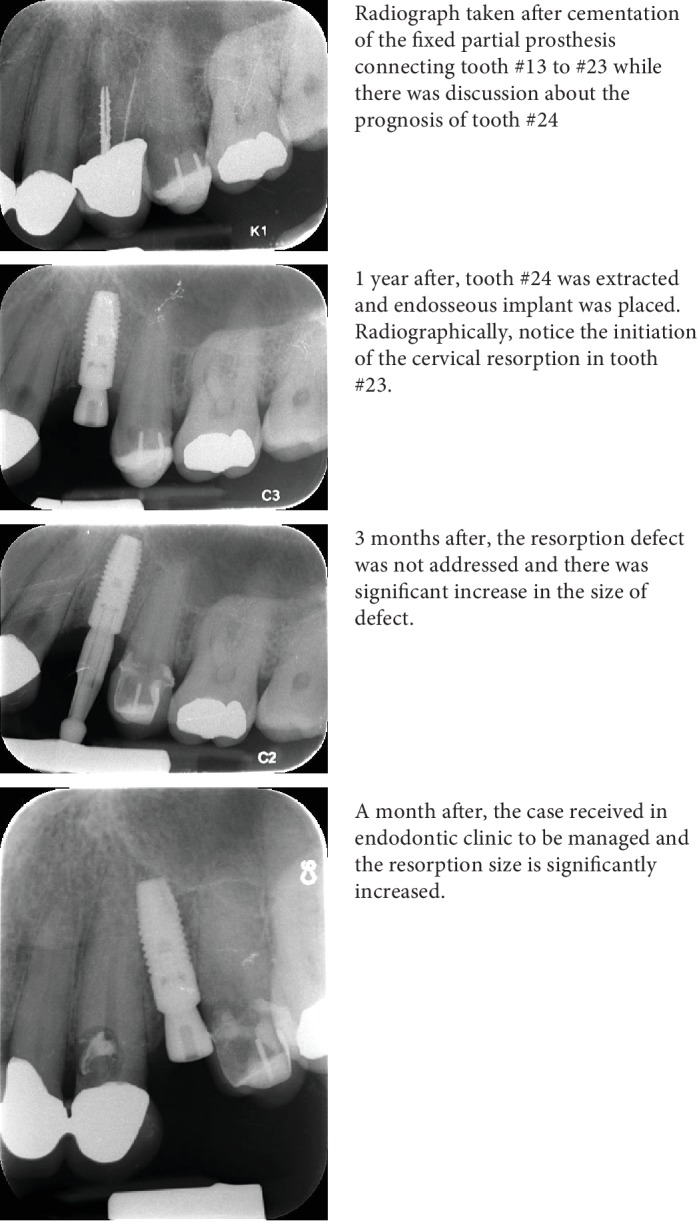
Chronological radiographic evaluation of the resorptive defect in tooth #23.

The ECR defect is characterized by root resorption in cervical third, which can progress rapidly. This patient was diagnosed clinically and intraoperatively by chance during a periodontal surgery in which a granulation tissue covering the whole palatal side of the coronal third and it was removed. However, in retrospection, radiographic progression of the resorptive defect was evident, as seen in Figure 4.

The management of this case involved a two-step procedure, which started with the nonsurgical approach and ended with surgery. We used dental amalgam as a post and core buildup material, followed by Geristore®. These materials had beneficial properties, which were well suited to this case, as shown by the recall visits.

The use of dental amalgam has been minimized or prohibited in some countries, mainly because of its potential health hazards, caused by mercury release [6]. We countered this drawback, by covering it with a high-sealing, periodontally compatible restorative material, which will also promote tissue attachment and maintain the proper root contour [7]. Geristore® even showed advantageous properties at inflammatory cytokine expression levels [8]. The advantages of amalgam over resin-bonded restorations include the ease of manipulation, good seal, and lower cost. Its principal property that informed our clinical decision was the technique insensitivity, as contamination is expected when dealing with such a large defect. Therefore, we could complete the nonsurgical root canal treatment in a single visit.

Surgical exposure of the defect permitted direct observation of the resorptive lesion. Cone beam computed tomography (CBCT) imaging can reveal such lesions preoperatively, thus aiding in treatment planning and management [9]. Limited access to CBCT at that time prevented its use in this case. We strongly recommend performing CBCT before treatment.
